# Fingerprint profiling for quality evaluation and the related biological activity analysis of polysaccharides from Liuweizhiji Gegen-Sangshen beverage

**DOI:** 10.3389/fnut.2024.1431518

**Published:** 2024-07-08

**Authors:** Shulin Wei, Mingxing Li, Long Zhao, Tiangang Wang, Ke Wu, Jiayue Yang, Mingyun Tang, Yueshui Zhao, Jing Shen, Fukuan Du, Yu Chen, Shuai Deng, Zhangang Xiao, Mei Wei, Zhi Li, Xu Wu

**Affiliations:** ^1^Cell Therapy & Cell Drugs of Luzhou Key Laboratory, Department of Pharmacology, School of Pharmacy, Southwest Medical University, Luzhou, China; ^2^South Sichuan Institute of Translational Medicine, Luzhou, China; ^3^Department of Spleen and Stomach Diseases, The Affiliated Traditional Chinese Medicine Hospital, Southwest Medical University, Luzhou, China; ^4^The Key Laboratory of Integrated Traditional Chinese and Western Medicine for Prevention and Treatment of Digestive System Diseases of Luzhou City, The Affiliated Traditional Chinese Medicine Hospital, Southwest Medical University, Luzhou, China

**Keywords:** Gegen-Sangshen beverage, polysaccharide, fingerprint, anti-inflammation, *Lactobacillus*, *Bifidobacterium*

## Abstract

**Introduction:**

Liuweizhiji Gegen-Sangshen beverage (LGS) is popular in China, which has been used for alleviating alcohol-mediated discomfort and preventing alcoholic liver disease (ALD). This beverage is consisted of six herbal components that are known as functional foods and fruits. LGS is rich in polysaccharides, however, the activity and quality evaluation of LGS-derived polysaccharides remain unexplored. The purpose of this study is thus to establish a comprehensive quality control methodology for the assessment of LGS polysaccharides (LGSP) and to further explore the anti-oxidant, anti-inflammatory as well as prebiotic effect of LGSP.

**Methods:**

LGSP was extracted, followed by analysis of molecular weight distribution, monosaccharide content and structural characterization via integrating the application of high-performance size exclusion chromatography (HPSEC), 1-phenyl-3-methyl-5-pyrazolone-HPLC (PMP-HPLC), fourier transform infrared spectroscopy (FT-IR) as well as nuclear magnetic resonance spectroscopy (NMR) techniques. The anti-oxidation activity of LGSP was determined by DPPH, ABTS, hydroxyl radical scavenging capacity and total antioxidant capacity. The anti-inflammation of LGSP were assessed on the RAW 264.7 cells. The effect of LGSP on growth of Lactobacillus, Bifidobacterium bifidum and Bifidobacterium adolescentis was evaluated.

**Results:**

The results demonstrated that LGSP had two molecular weight distribution peaks, with the average molecular weights of (6.569 ± 0.12) × 10^4^ Da and (4.641 ± 0.30) × 10^4^ Da. LGSP was composed of 8 monosaccharides, with galacturonic acid, glucose rhamnose and galactose representing the highest molar ratios. Homogalacturonic acid (HG) type and rhamnosegalacturonic acid glycans I (RG-I) type and α-1,4-glucan were present in LGSP. LGSP concentration in LGS was 17.94 ± 0.28 mg/mL. Furthermore, fingerprint analysis combined with composition quantification of 10 batches of LGSP demonstrated that there was a high similarity among batches. Notably, LGSP exhibited anti-oxidant effect and inhibited expressions of pro-inflammatory factors (TNF-α and IL-6) in LPS-stimulated RAW 264.7 cells. In addition, LGSP remarkably promoted the proliferation of probiotics Lactobacillus, Bifidobacterium bifidum and Bifidobacterium adolescentis, showing good prebiotic activity.

**Discussion:**

The results of present study would be of help to gain the understanding of structure–activity relationship of LGSP, provide a reference for quality evaluation of bioactive LGSP, and facilitate development of unique health and functional products in the future.

## Introduction

Liuweizhiji^®^ Gegen-Sangshen beverage (LGS), consisting of six functional and herbal foods, including Puerariae lobatae radix (The dried radix of *Pueraria lobata* (Willd.) Ohwi), Hoveniae semen (The dried fruit and seed of *Hovenia dulcis* Thunb), Imperatae rhizoma (The dried rhizome of *Imperata cylindrica* Beauv. var. major (Nees) C. E. Hubb), Crataegi fructus (The dried fructus of *Crataegus pinnatifida* Bge.), Mori fructus (The dried fructus of *Morus alba* L.) and Canarli fructus (The dried fructus of *Canarium album* Raeusch.), is available in market and is popular in southwest China, which has been used for alleviating alcohol-mediated discomfort and preventing alcoholic liver disease (ALD) (1). This functional drink and its components have been traditionally used to treat digestive tract disorders including liver dysfunction. The main components of LGS included flavonoids, polyphenols, organic acids, as well as the macromolecular polysaccharides. It was demonstrated that some flavonoids and organic acids, including puerarin and its derivatives, vitexin, rutin and gallic acid, showed anti-oxidant, anti-inflammatory and gut microbiota regulating activities and contributed to the ALD alleviating effect of LGS (2–5). Current studies on active ingredients of LGS are mainly focused on the small-molecule components (1), however, the polysaccharide fraction is largely unexplored. The activity and quality control of polysaccharides from LGS necessitate further studies.

Polysaccharides derived from varied sources exhibit potent biological effects, such as antioxidant, antitumor, immunomodulatory, hypoglycemic, and hepatoprotective properties (6–8). Previously, it was reported that *Pueraria lobata* polysaccharides, possessed antioxidant and immunomodulatory activities, and regulated gut microbiome (9, 10). Polysaccharides from Crataegi fructus modulated lipid metabolism and restored the imbalance of intestinal microbiota (11). Similarly, polysaccharides from Mori fructus showed anti-inflammation, antioxidant, and hepatoprotective properties (12, 13). Biologically active polysaccharides were also identified from rest of the three herbs (14–16). It is thus speculated that polysaccharides are an important fraction of LGS. However, after complex processing and manufacturing, the structure of LGS polysaccharides (LGSP) may be altered significantly. Moreover, whether LGSP exerts biological effects (including antioxidant, anti-inflammatory and prebiotic effects) related to the anti-ALD effect of LGS remains unclear.

Owing to the complicate structure and large molecular weight of polysaccharides, quality control of polysaccharides is considered difficult. Natural plant polysaccharides are complex carbohydrate polymers consisting of varied long-chain monosaccharide units, and the quantification of a single marker substance is difficult and cannot specifically reflect the whole structural characteristics of polysaccharides (17). Integrated techniques to evaluate polysaccharide quality from different aspects, including monosaccharide contents, molecular weight, and distinctive structures, is thus of great significance (18). Multiple fingerprinting using spectroscopic and chromatographic methods as well as chemometrics have been proposed for the quality assessment (19). The determination of monosaccharide composition is indispensable in polysaccharide fingerprinting, and the commonly used detection methods include 1-phenyl-3-methyl-5-pyrazolone-HPLC (PMP-HPLC), gas chromatography–mass spectrometry (GC–MS), high-performance size exclusion chromatography (HPSEC), and high performance anion exchange chromatography-pulsed amperometric detector (HPAEC-PAD) (20, 21). In addition, gel permeation chromatography has been used for molecular weight distribution fingerprinting (22). Structural characteristic fingerprinting by fourier transform infrared spectroscopy (FT-IR) as well as nuclear magnetic resonance spectroscopy (NMR) analysis affords sufficient structural information of polysaccharides (21). Thus, multi-fingerprinting analysis combined with quantitative analysis present as an efficient and effective method for evaluation of the quality consistency of polysaccharides, especially those derived from complex herbal preparations with potential batch-to-batch differences.

Based on the limitations on current studies of LGSP, the purpose of this study is to establish a multiple-fingerprint-based methodology for quality control assessment of LGSP and to investigate the related biological activity of LGSP. In this study, the LGSP were extracted from LGS, and multi-fingerprint analysis based on HPSEC, PMP-HPLC, FT-IR, and NMR were performed to analyze the molecular weight, the types and proportions of constituent monosaccharides, the types of characteristic functional groups and glycosidic bonds of LGSP from 10 batches of samples. The contents of total polysaccharides, total acidic sugars, and the content distribution of different molecular weight components were also investigated. Eventually, the antioxidant, anti-inflammatory and prebiotic activities of LGSP were analyzed by *in vitro* assays. The results of current study would gain better understanding towards the structure of LGSP, provide basis for the quality evaluation, and support for the functional use of the active polysaccharide fraction of LGS.

## Materials and methods

### Materials and chemicals

Amyloglucosidase, heat-stabilized α-amylase, D-glucuronic acid, L-rhamnose monohydrate, D-(+)-glucose, D-(+)-mannose, L-arabinose, D-galacturonic acid, KBr (AR grade), bovine serum albumin (BSA) and coomassie brilliant blue were obtained via Shanghai Yuanye Bio-Technology (Shanghai, China). D-xylose and D-galactose were provided by Shanghai Aladdin Biochemical Technology (Shanghai, China). PMP, trifluoracetic acid (TFA) and dextran were obtained from Sigma-Aldrich (St. Louis, MO, United States). Glucose, lipopolysaccharide (LPS), 5-diphenyltetrazolium bromide (MTT) and dimethyl sulfoxide (DMSO) were obtained from Shanghai Macklin Biochemical Technology Co., Ltd. (Shanghai, China). MRS medium without glucose was got from Shandong Tuopu Biol-engineering (Shandong, China). DMEM culture medium and fetal bovine serum (FBS) were purchased from Shanghai Xiaopeng Biotechnology Co., Ltd. (Shanghai, China). Primers were purchased from Tsingke Biotechnology Co., Ltd. (Beijing, China). Acetonitrile and methanol (HPLC grade) were obtained from Chron Chemicals (Chengdu, China). Ultra-pure water was prepared by Milli-Q (Merck, Germany).

*Lactobacillus* (CICC 6269), *Bifidobacterium bifidum* (CICC 6071), *Bifidobacterium adolescentis* (CICC 6070) and *Escherichia coli* (CICC 10899) were purchased from the China Center of Industrial Culture Collection (CICC, Beijing, China). Under anaerobic condition at 37°C, *Lactobacillus* was cultured in MRS medium, *E. coli* was cultured in LB medium, while *B. bifidum* and *B. adolescentis* were cultured in CM0233 medium.

RAW264.7 macrophages were obtained from Cyagen Biotechnology Co., Ltd. (Guangdong, China). Macrophages were inoculated into DMEM medium supplemented with 10% FBS, 100 U/mL penicillin, and 100 U/mL streptomycin. The cells were then placed at 37°C in an incubator with 5% CO_2_ humidified atmosphere.

A total of 10 batches of LGS (S1-S10) were supplied by Sichuan Tongyou Life Health Technology Co., Ltd. (Sichuan, China). Voucher specimens (Batch Number: 230101–230,110) were deposited at School of Pharmacy, Southwest Medical University, Luzhou, China.

### Extraction and purification of LGSP

An aliquot of 30 mL LGS was mixed with 40 mL of ultrapure water. Thermostable α-amylase was added at 10 U/mL, maintaining at 70°C for 8 h. After cooling down, amyloglucosidase was added at 10 U/mL for removing starch. The reaction was taken at 59°C for 12 h, and finally heated to 95°C for 30 min to inactivate the enzyme. After further centrifugation at 4000 × g for 15 min, 4 volumes of alcohol (95%, v/v) were mixed with the supernatant for precipitating crude polysaccharides. Next, crude polysaccharides were washed and redissolved and freeze-dried to obtain LGSP, which was stored at 4°C.

### HPSEC fingerprint

The molecular weight and distribution of LGSP were analyzed by HPSEC connecting to a multi-angle laser light scattering and differential index detector (HPSEC-MALLS-RID), and molecular weight fingerprint of polysaccharides of LGS was established. The column used was Shodex OHpak SB-804 HQ column (8.0 × 300 mm). The 0.9% NaCl in water served as the mobile phase. The flow rate was 0.5 mL/min. The injection volume was set to 100 μL.

### PMP-HPLC fingerprint

#### Complete acid hydrolysis

The complete acid hydrolysis of LGSP analyzed by PMP derivatization was conducted as previously reported (23). In brief, 6 mg of LGSP is precisely weighed and dissolved in 1 mL of ultrapure water and hydrolyzed by with 1 mL of 4 mM TFA at 95°C for 12 h. After complete removal of TFA residues under a vacuum evaporator, it is dissolved in 1 mL of ultrapure water for follow-up PMP derivatization. The monosaccharide standard solutions (0.5 mg/mL; including galacturonic acid, mannose, rhamnose, glucose, glucuronic acid, galactose, xylose, and arabinose) or the hydrolysate samples (50 μL; 6 mg/mL) were, respectively, added with 50 μL of sodium hydroxide (0.6 mM) and 100 μL of PMP solution (0.5 mM) for reaction for 100 min at 70°C. After neutralization with 30 μL of 0.3 mM HCl and removing PMP by chloroform extraction, the derivatization solution was filtered for further analysis.

Derivatized samples were analyzed on a Thermo UHPLC-3000 SL system with a ZORBAX Eclipse XDB-C18 column (150 mm × 4.6 mm, 5 μm) equipped with a diode array detector (DAD). The column temperature was controlled at 35°C. Isocratic elution was performed using the mixed mobile phase of A (0.1 M PBS, pH = 6.7) and B (acetonitrile) with a *v*/*v* ratio of 83.5:16.5. The flow rate was 1.0 mL/min. Injection volume was 20 μL. The detection wavelength was set at 245 nm for DAD analysis.

#### Partial acid hydrolysis

The partial acid hydrolysis of LGSP was conducted by reaction of 0.5 mM TFA with 6 mg/mL LGSP at 95°C for 5 h. The other procedures including PMP derivatization and ultra-high performance liquid chromatography (UHPLC) analysis were identical to those for complete acid hydrolysis.

### FT-IR fingerprint

The FT-IR spectra (ranged 4,000–500 cm^−1^) of LGSP were acquired on a PerkinElmer Spectrum 3 FT-IR spectrometer.

### NMR fingerprint

The LGSP samples were dissolved in D_2_O. The one-dimensional hydrogen spectra (^1^H) and one-dimensional carbon spectra (^13^C) of LGSP were analyzed by a Bruker Ascend 600 MHz spectrometer using a z-gradient probe. The information acquisition temperature was 25°C, the carbon frequency was 150.90 MHz, and the proton frequency was 600.13 MHz.

### Chemical composition analysis

Using the following formula, the extraction rate of LGSP was calculated: yields (%) = (weight of LGSP / volume of LGS) × 100%. Components of LGSP, such as total polysaccharides, total proteins and total uronic acids, were determined using the colorimetric method. In brief, total contents of polysaccharides were quantified using phenol-sulfuric acid method with galactose and galacturonic acid as standards. Using galacturonic acid as the standard, the m-hydroxybiphenyl method was applied to detect the quantity of uronic acid. The protein contents were analyzed by the Bradford method with BSA used as the standard.

### Activity of LGSP

#### Antioxidant activity

The antioxidant activity of LGSP was assessed by the DPPH free radical scavenging assay, the hydroxyl radical scavenging assay, the ABTS free radical scavenging assay and the total antioxidant capacity test according to the kit manufacturer’s instructions.

#### Anti-inflammatory effect

The anti-inflammation of LGSP were assessed on the RAW 264.7 cells. The impact of LGSP on macrophage proliferation was firstly detected using the MTT method. For the anti-inflammation study, pre-cultured RAW 264.7 cells were collected and seeded in 6-well plates (6 × 10^5^/mL). LPS (1 μg/mL) was added for induction for 4 h, and then the cell morphology changes were observed before adding the LGSP (0–200 μg/mL). After 24 h of drug treatment, cells were collected, and total RNA was extracted with Trizol reagent. Reverse transcription into cDNA was conducted by using a reverse transcription kit (Vazyme), and GAPDH was used as the internal control. The mRNA expression level was determined by quantitative PCR (qPCR). The primer sequences were as follows: *GAPDH* (forward, AGGTCGGTGTGAACGGATTTG; reverse, TGTAGACCATGTAGTTGAGGTCA); *IL-6* (forward, TCTATACCACTTCACAAGTCGGA; reverse, CGATCACCCCGAAGTTCAGTAG); *TNF-a* (forward, CAGGCGGTGCCTATGTCTC; reverse, CGATCACCCCGAAGTTCAGTAG). The culture supernatants were used for determination of the concentrations of IL-6 and TNF-α using the enzyme linked immunosorbent assay (ELISA) kits (Jiangsu Meibiao Biotechnology Co., Ltd., Jiangsu, China).

#### Prebiotic activity

During experimentation, sugar-free culture medium was used. In order to evaluate whether LGSP had an effect on bacterial growth, LGSP at 4000 mg/L was supplemented as the carbon source. Glucose added at 4000 mg/L was used as a positive control. The bacterial growth was recorded at the time points of 0, 4, 8, 12, and 24 h, respectively, through determining the optical density (OD) at 600 nm with a spectrophotometer. In addition, to test the particularity of LGSP in promoting the growth of beneficial bacteria, the impact of LGSP on the growth of *E. coli* was further investigated.

### Statistical analysis

GraphPad Prism 6.0 was used for data analysis. All data are displayed as mean ± standard deviation (SD). Statistical analysis was carried out using one-way analysis of variance (ANOVA) followed by Tukey’s test.

## Results

### Quality evaluation

#### Fingerprint based on molecular weight distribution

**Figure 1:**
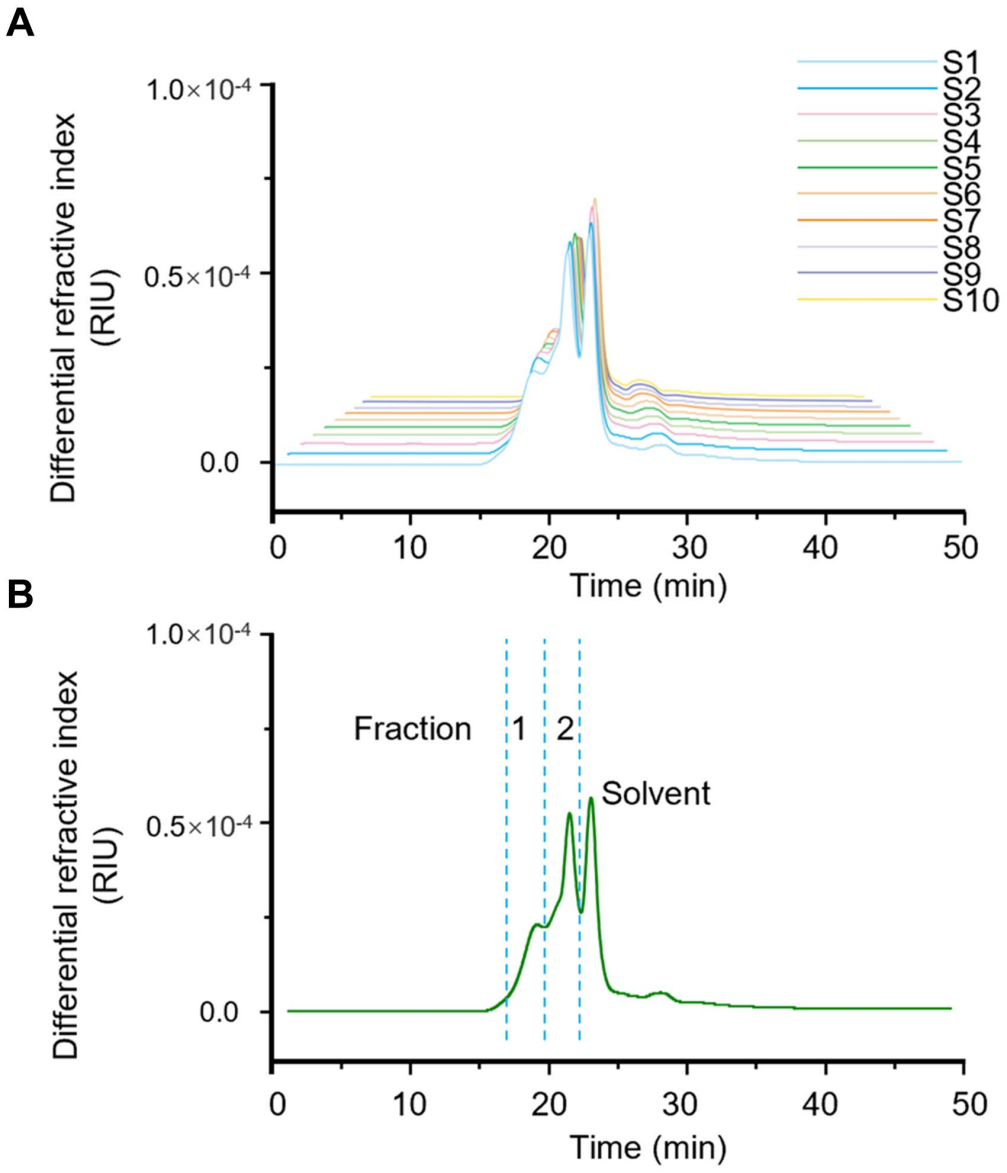
Fingerprints of molecular weight distribution of LGSP by HPSEC-MALLS-RID. **(A)** The analysis of different batches (S1-S10) of LGSP. **(B)** The generated standard fingerprint for molecular weight distribution of LGSP which contained two major fractions 1 and 2. The solvent peak was resulted from the dissolving reagent of LGSP.

HPSEC fingerprint of 10 batches of LGSP was first conducted to reveal the characteristics of molecular weight distribution. The results are shown in Table 1 and Figure 1. The solvent background peaks that appeared after 21 min were removed, and the 10 batches of LGSP had similar spectra and molecular weight distributions, which contained two molecular weight distribution peaks, namely the fraction 1 and fraction 2.

**Table 1:** Molecular weight distribution of polysaccharides in different batches of LGSP.

No.	Fraction 1 M_w_ × 10^4^ (Da)	Relative peak areas (%)	M_w_/M_n_	Fraction 2 M_w_ × 10^4^ (Da)	Relative peak areas (%)	M_w_/M_n_
S1	6.436	32.1	1.168	4.242	67.9	1.092
S2	6.443	33.2	1.189	4.068	66.8	1.109
S3	6.763	32.5	1.162	4.880	67.5	1.076
S4	6.531	32.8	1.150	4.881	67.2	1.075
S5	6.667	31.4	1.154	4.886	68.6	1.088
S6	6.689	33.7	1.159	4.681	66.3	1.082
S7	6.570	30.8	1.165	4.353	69.2	1.115
S8	6.621	32.1	1.169	4.851	67.9	1.076
S9	6.387	33.1	1.155	4.930	66.9	1.094
S10	6.583	32.8	1.163	4.640	67.2	1.089
Average	6.569	32.45	1.163	4.641	67.55	1.090

M_w_, weight-average molecular weight; M_n_, number-average molecular weight; M_w_/M_n_, index of molecular weight distribution.

The molecular weight of fraction 1 ranged from 6.387 × 10^4^ Da to 6.763 × 10^4^ Da, with the average molecular weight of 6.569 × 10^4^ Da. It is worth noting that sample 3 had the highest molecular weight of fraction 1, while sample 9 had the lowest molecular weight of fraction 1. The relative proportion of fraction 1 accounted for 30.8–33.7% (average: 32.45%) of the total peak area. The molecular weight range of fraction 2 of different batches of LGSP was 4.068 × 10^4^ Da – 4.930 × 10^4^ Da, and the average molecular weight was 4.641 × 10^4^ Da. The sample 9 had the highest molecular weight of fraction 2 and sample 2 had the lowest molecular weight. The relative proportion of the peak area of fraction 2 was 66.3–69.2%, with the average proportion of 67.55%. The peak area ratio of fraction 2 in different batches of LGSP was about twice that of fraction 1.

#### Fingerprint based on monosaccharide composition

**Figure 2:**
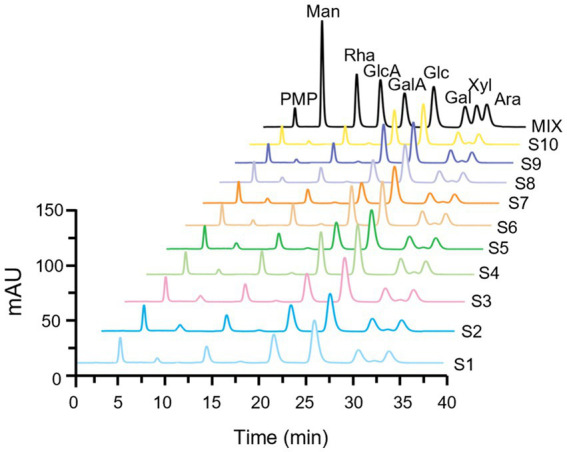
Fingerprints of monosaccharide composition of different batches (S1-S10) of LGSP by PMP-HPLC after complete acid hydrolysis. MIX represents the chromatogram of mixed monosaccharide standards. Ara, arabinose; Gal, galactose; GalA, galacturonic acid; Glc, glucose; GlcA, glucuronic acid; Man, mannose; Rha, rhamnose; Xyl, xylose.

Monosaccharides serve as the fundamental building blocks of polysaccharides, playing a crucial role in identifying their structural characteristics. The monosaccharide composition and the molar percentage among different batches of LGSP were analyzed by complete hydrolysis followed by HPLC analysis after pre-column PMP derivatization. As shown in Figure 2, the monosaccharide compositions of different batches of LGSP were basically the same, which were composed of 8 monosaccharide components: mannose (Man), glucose (Glc), galactose (Gal), xylose (Xyl), rhamnose (Rha), arabinose (Ara), glucuronic acid (GlcA), and galacturonic acid (GalA). The molar ratios of 10 batches of LGSP are shown in Table 2. The molar percentages of different batches of LGSP were similar, among which Ara, Glc, GalA, Gal and Rha were the predominant monosaccharides.

**Table 2:** Monosaccharide composition and molar ratio of different batches of LGSP by complete acid hydrolysis.

No.	Man	Rha	GlcA	GalA	Glc	Gal	Xyl	Ara	MR1	MR2
S1	0.0132	0.1141	0.009	0.2499	0.3767	0.1141	0.0202	0.1028	2.19	1.90
S2	0.0438	0.1186	0.0082	0.2397	0.3481	0.1183	0.0197	0.1035	2.02	1.87
S3	0.0356	0.1172	0.008	0.2348	0.3696	0.1142	0.0196	0.101	2.00	1.84
S4	0.0183	0.111	0.0094	0.269	0.3487	0.116	0.018	0.1094	2.42	2.03
S5	0.0837	0.1086	0.009	0.2313	0.3414	0.1117	0.0177	0.0967	2.13	1.92
S6	0.0185	0.1118	0.0093	0.2813	0.3397	0.1145	0.0185	0.1065	2.52	1.98
S7	0.0609	0.1153	0.0078	0.2125	0.3824	0.1074	0.0184	0.0953	1.84	1.76
S8	0.0522	0.1167	0.0078	0.2197	0.3726	0.114	0.0191	0.098	1.88	1.82
S9	0.0167	0.1112	0.0093	0.2947	0.3375	0.11	0.0178	0.1029	2.65	1.91
S10	0.0178	0.1106	0.0091	0.2725	0.3534	0.1121	0.0177	0.1067	2.46	1.98
Average	0.0361	0.1135	0.0087	0.2505	0.3570	0.1132	0.0187	0.1023	2.21	1.90

Ara, arabinose; Gal, galactose; GalA, galacturonic acid; Glc, glucose; GlcA, glucuronic acid; Man, mannose; Rha, rhamnose; Xyl, xylose; MR1, the molar ratio of GalA/Rha; MR2, the molar ratio of (Ara + Gal)/Rha.

In general, GalA, Gal, Ara, Rha, and GlcA are the typical monosaccharides of homogalacturonic acid (HG) type and rhamnosegalacturonic acid glycans I (RG-I) type polysaccharides. The molar ratio (MR) of monosaccharides can unveil the structural characteristics of polysaccharides. MR1 is the ratio of GalA/Rha, indicating the proportion of HG to RG-I domains. In addition, MR2 is the ratio of (Ara + Gal)/Rha, which reflects the degree of branching of RG-I. Higher values indicate that the RG-I region contains more or longer side chains. The results showed that the MR1 of different batches of LGSP ranged from 1.84 to 2.65, while MR2 ranged from 1.76 to 2.03. Therefore, according to the MR values, HG and RG-I pectin polysaccharides were present in the LGSP. In addition, although the samples were destarched with amylase and saccharification enzyme, Glc was still present in the LGSP, which may be resistant starch that could not be removed by the two enzymes. This indicated that different batches of LGSP also contained dextran.

#### Fingerprint based on controllable partial acid hydrolysis

Partial acid hydrolysis can simplify the complex structure of polysaccharides and degrade them into oligosaccharides with different degrees of polymerization. Unlike complete acid hydrolysis, partial acid hydrolysis is different in acid concentration or treatment time, which leads to different final hydrolysate products, and polysaccharide structure can be analyzed from different aspects (24). Thus, controllable partial acid hydrolysis of LGSP was carried out using low-concentration TFA, and the analysis of the controllable partial acid hydrolysate and the molar percentage of monosaccharide compositions were analyzed by HPLC after pre-column PMP derivatization. Fingerprints for both complete and partial acid hydrolysates would provide a more comprehensive information for chemical compositions of LGSP.

**Figure 3:**
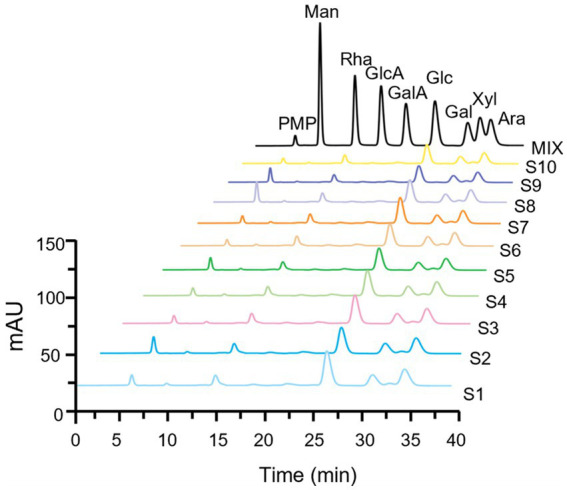
Fingerprints of monosaccharide composition of different batches (S1-S10) of LGSP by PMP-HPLC after controllable partial acid hydrolysis. MIX represents the chromatogram of mixed monosaccharide standards. Ara, arabinose; Gal, galactose; GalA, galacturonic acid; Glc, glucose; GlcA, glucuronic acid; Man, mannose; Rha, rhamnose; Xyl, xylose.

As shown in Figure 3, the monosaccharide compositions in the partial hydrolysate of LGSP were generally similar, which were composed of five monosaccharide components: Rha, Glc, Gal, Xyl and Ara. This indicated that the controllable partial acid hydrolysis did not destroy the HG pectin domain in the LGSP. In addition, the molar compositions of monosaccharides in the partial acid hydrolysate of different batches of LGSP were slightly different, and the composition of each monosaccharide in the partial hydrolysate of LGSP is shown in Table 3. The main monosaccharides in the partial acid hydrolysate of LGSP were Rha, Glc, Gal and Ara, among which the molar percentage of Rha was 10.46–14.04% averaged at 12.14%. The molar percentage of Glc was 41.23–48.65% averaged at 44.39%. The molar percentage of Gal was 14.92–17.36% averaged at 16.10%. The molar mass percentage of Ara was 22.86–25.43% averaged at 24.24%. Notably, the MR2 ratio of different batches of LGSP was 2.88–3.78, and the average value was 3.35.

**Table 3:** The composition and proportion of monosaccharides in the partial acid hydrolysate of different batches of LGSP.

No.	Rha	Glc	Gal	Xyl	Ara	MR2
S1	0.1046	0.4865	0.1506	0.0283	0.2300	3.64
S2	0.1187	0.4299	0.1658	0.0313	0.2543	3.54
S3	0.1062	0.4616	0.1600	0.0305	0.2417	3.78
S4	0.1179	0.4415	0.1662	0.0301	0.2443	3.48
S5	0.1157	0.4568	0.1582	0.0306	0.2388	3.43
S6	0.1311	0.4134	0.1736	0.0337	0.2482	3.22
S7	0.1227	0.4695	0.1492	0.0299	0.2286	3.08
S8	0.1193	0.4434	0.1592	0.0329	0.2452	3.39
S9	0.1378	0.4123	0.1669	0.0335	0.2495	3.02
S10	0.1404	0.4245	0.1606	0.0312	0.2433	2.88
Average	0.1214	0.4439	0.1610	0.0312	0.2424	3.35

Ara, arabinose; Gal, galactose; Glc, glucose; Rha, rhamnose; Xyl, xylose; MR2, molar ratio of (Ara + Gal)/Rha.

#### Fingerprint based on FT-IR

**Figure 4:**
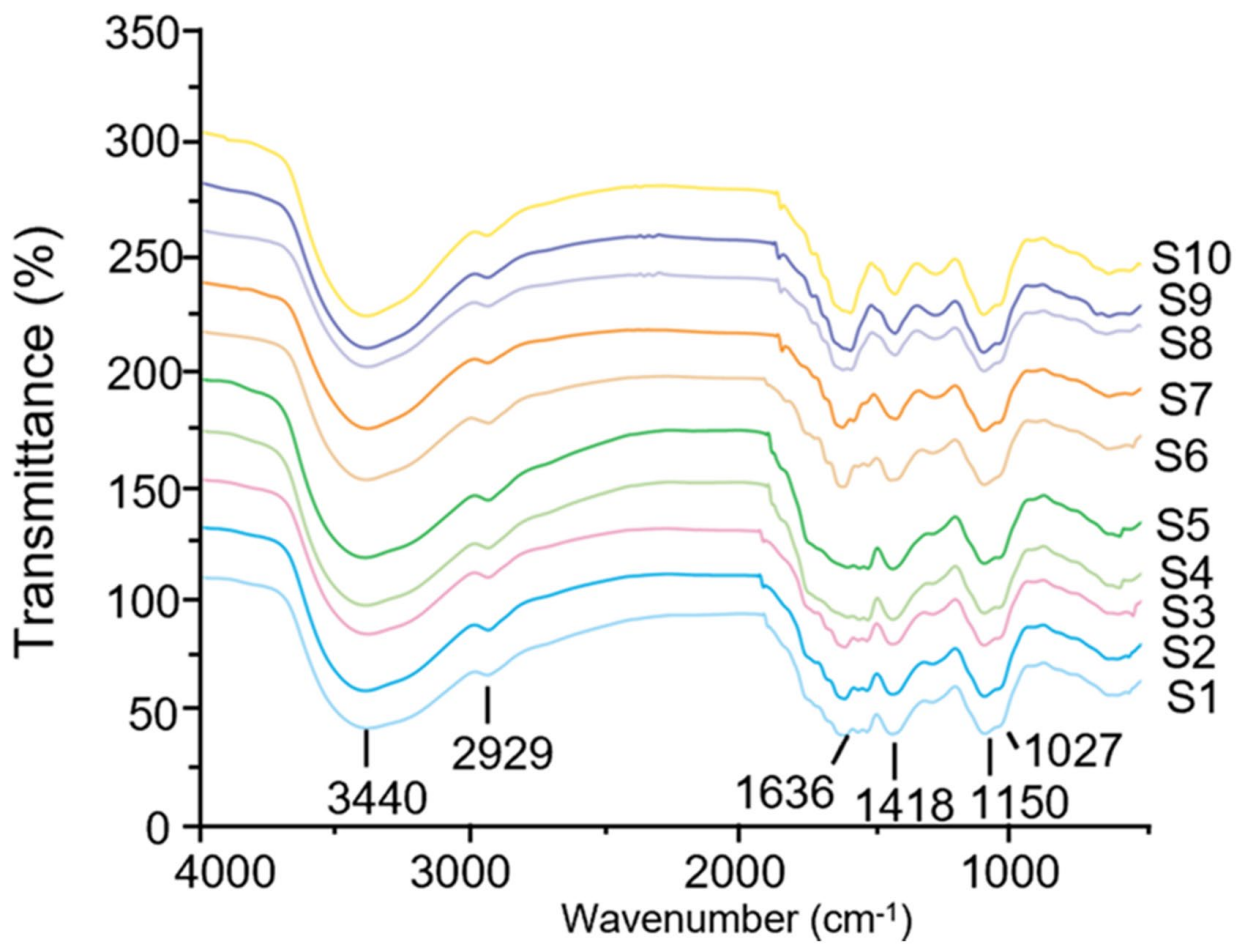
FT-IR fingerprints of different batches (S1-S10) of LGSP.

The FT-IR spectrometer was used to record the infrared spectra in the wavelength range of 4,000–500 cm^−1^, and the FT-IR fingerprint of different batches of LGSP was established. As shown in Figure 4, the FT-IR spectra of different batches of LGSP were similar. The vibrational region shown between 3,200 cm^−1^ and 3,600 cm^−1^ was the featured band of the hydroxyl groups, and the peak at 3400 cm^−1^ indicated the stretching vibration of hydroxyl group, representing the presence of hydroxyl groups. The fluctuations in the range of 3,000 cm^−1^ -2800 cm^−1^ (2,929 cm^−1^) were the C-H absorption peaks, which included the stretching vibrations of CH, CH_2_, and CH_3_. The strong peak at 1636 cm^−1^ was the asymmetric stretching vibration of C=O in the carboxylic acid group, which represented the presence of uronic acid. The vibration at 1418 cm^−1^ was attributed to the bending vibration of C-H or O-H, and the vibration at 1150 cm^−1^ was an asymmetrical C-O-C vibration, representing the presence of a methoxy group. In addition, the vibrational range (1,000 cm^−1^ -1200 cm^−1^) was caused by the vibration of the ester glycan group (CO-C) and the C-O-H of the pyranose ring, which proved that the LGSP contained pyranonose.

#### Fingerprint based on NMR

**Figure 5:**
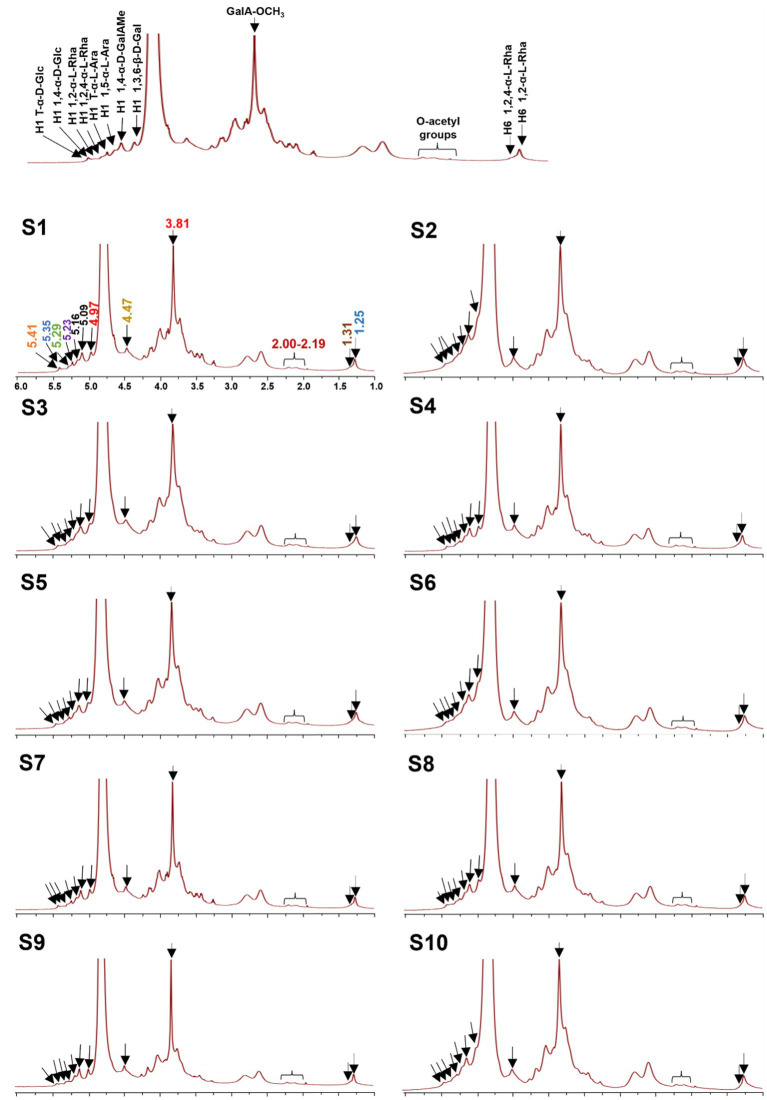
^1^H NMR fingerprints of different batches (S1-S10) of LGSP with structural assignments of key signals.

**Figure 6:**
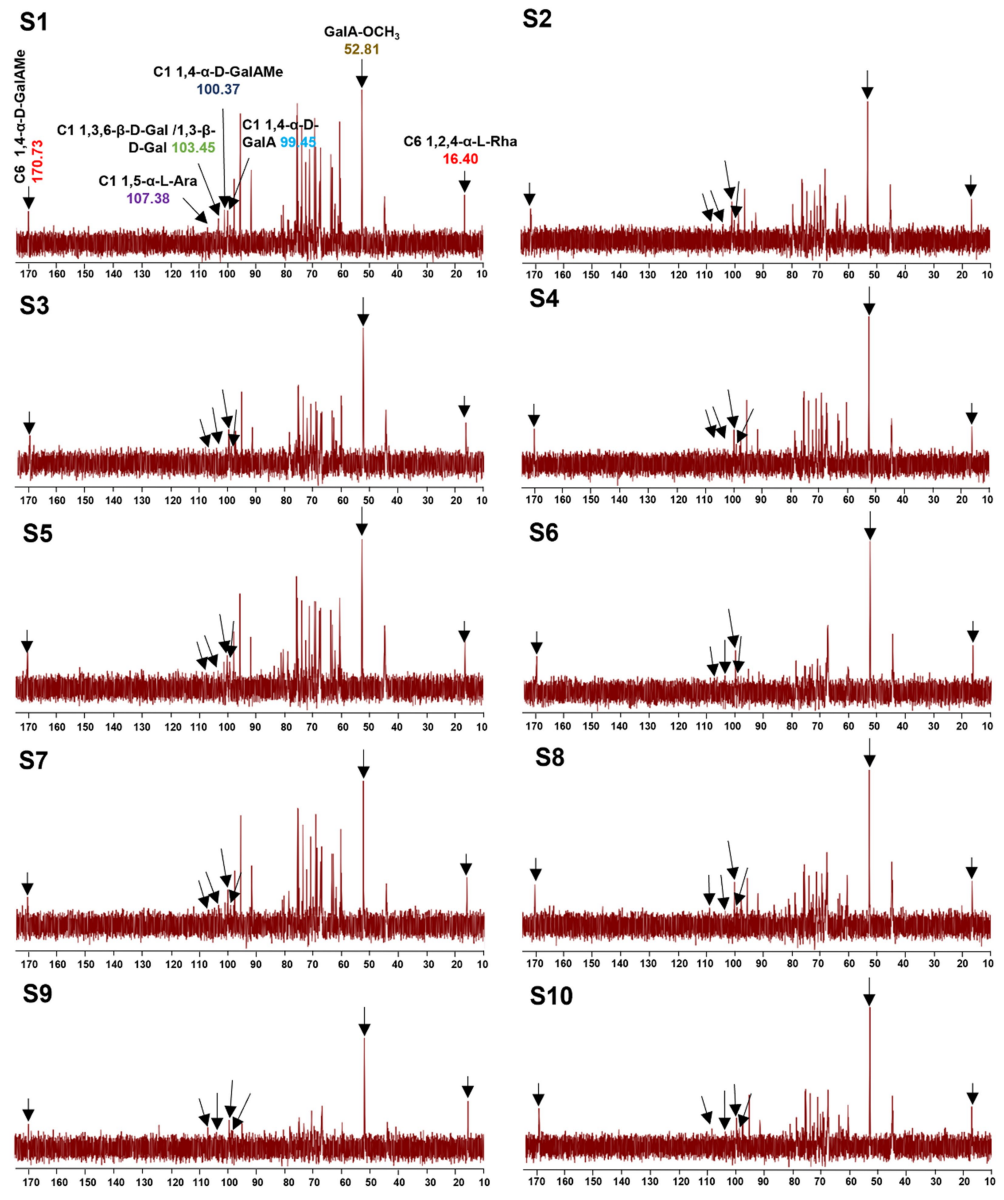
^13^C NMR fingerprints of different batches (S1-S10) of LGSP with structural assignments of key signals.

To better understand the precise structure of the LGSP, the ^1^H and ^13^C NMR spectra of 10 batches of LGSP were measured and analyzed by Bruker NMR spectroscopy (Figures 5, 6). The ^1^H and ^13^C NMR spectra of the 10 batches of LGSP were similar, indicating that they had similar primary chemical structures.

Typical signals of pectins (HG and RG-I) as well as α-glucan, were observed from ^1^H and ^13^C NMR. For example, from the ^1^H spectra, the ^1^H NMR signals in the range of 5.09 to 5.29 ppm were attributed to the α-L-Ara and α-L-Rha residues. The signals in the range of 4.40 to 4.53 ppm were attributed to the β-D-Gal residue. The signals in the range of 4.97 ppm and 3.81 ppm were attributed to the GalAMe*p* residue. The presence of signals (5.41 and 5.35 ppm) suggested the presence of T-α-D-Glc and 1,4-α-D-Glc, which further confirmed the presence of resistant starch in the LGSP. The signals at 5.29 ppm and 5.23 ppm were corresponded to the presence of 1,2-α-L-Rha and 1,2,4-α-L-Rha, respectively, while the 5.16 ppm and 5.09 ppm ^1^H NMR signals were attributed to T-α-L-Ara and 1,5-α-L-Ara, respectively. The signal of 4.97 ppm was suggestive of the presence of 1,4-α-D-GalAMe*p*, while the signal at GalA-OCH_3_ was observed at 3.81 ppm. The signal at 4.47 ppm suggested the presence of 1,3,6-β-D-Gal. The signal at 2.00–2.09 ppm indicated the presence of the O-acetyl chemical group. Signals at 1.25 ppm and 1.31 ppm indicated the presence of 1,2-α-L-Rha and 1,2,4-α-L-Rha, respectively.

Based on ^13^C spectra, the signals at 170.73 and 100.37 ppm were due to the presence of 1,4-α-D-GalAMe*p*, while the signal of GalA-OCH_3_ was observed at 52.81 ppm. The signal at 107.38 ppm indicated the presence of 1,5-α-L-Ara, while the signal at 103.45 ppm suggested the presence of 1,3,6-β-D-Gal. The signal at 99.45 ppm indicated the 1,4-α-D-GalA, while the signal at 16.40 ppm indicated the 1,2,4-α-L-Rha. According to the data of monosaccharide composition analysis combined with NMR analysis, HG, RG-I and α-1,4-glucan were indeed found in 10 batches of LGSP.

#### Quantitative analysis

The total polysaccharides, extraction rate, total uronic acids and total protein contents of different batches of LGS are shown in Table 4. The results showed that the yield of LGSP ranged from 17.34 ± 0.01 mg/mL to 18.84 ± 0.53 mg/mL of LGS averaged at 17.94 ± 0.28 mg/mL. Different batches of LGSP contained a small amount of protein, with the content ranging from 2.72 to 3.39% averaged at 3.03%. The total polysaccharide content in different batches of LGSP ranged from 76.44 to 83.18%, and the average content was 79.42%. The total uronic acid content in different batches of LGSP ranged from 21.23 to 22.77%, and the average content was 22.02%.

**Table 4:** Total polysaccharides, total uronic acid, and total protein content of different batches of LGSP.

Batch	Extraction rate (mg/mL)	Polysaccharide (%)	Uronic acid (%)	Protein (%)
S1	17.43 ± 0.13	82.64 ± 0.68	21.23 ± 0.16	2.78 ± 0.02
S2	17.98 ± 0.49	83.18 ± 0.76	22.20 ± 0.17	3.39 ± 0.01
S3	17.91 ± 0.65	78.78 ± 0.45	21.33 ± 0.11	3.25 ± 0.08
S4	17.54 ± 0.15	76.44 ± 0.13	21.84 ± 0.05	3.19 ± 0.01
S5	17.34 ± 0.01	77.18 ± 0.29	21.99 ± 0.04	3.19 ± 0.04
S6	17.35 ± 0.17	80.97 ± 0.55	22.40 ± 0.11	2.96 ± 0.04
S7	18.68 ± 0.33	78.36 ± 0.44	22.24 ± 0.12	2.83 ± 0.03
S8	17.69 ± 0.17	76.86 ± 0.30	22.00 ± 0.27	2.72 ± 0.06
S9	18.84 ± 0.53	79.49 ± 1.76	22.77 ± 0.04	3.11 ± 0.08
S10	18.62 ± 0.13	80.31 ± 0.39	22.15 ± 0.06	2.89 ± 0.07
Average	17.94 ± 0.28	79.42 ± 0.58	22.02 ± 0.11	3.03 ± 0.04

### Activity evaluation

#### Anti-oxidant activity

**Figure 7:**
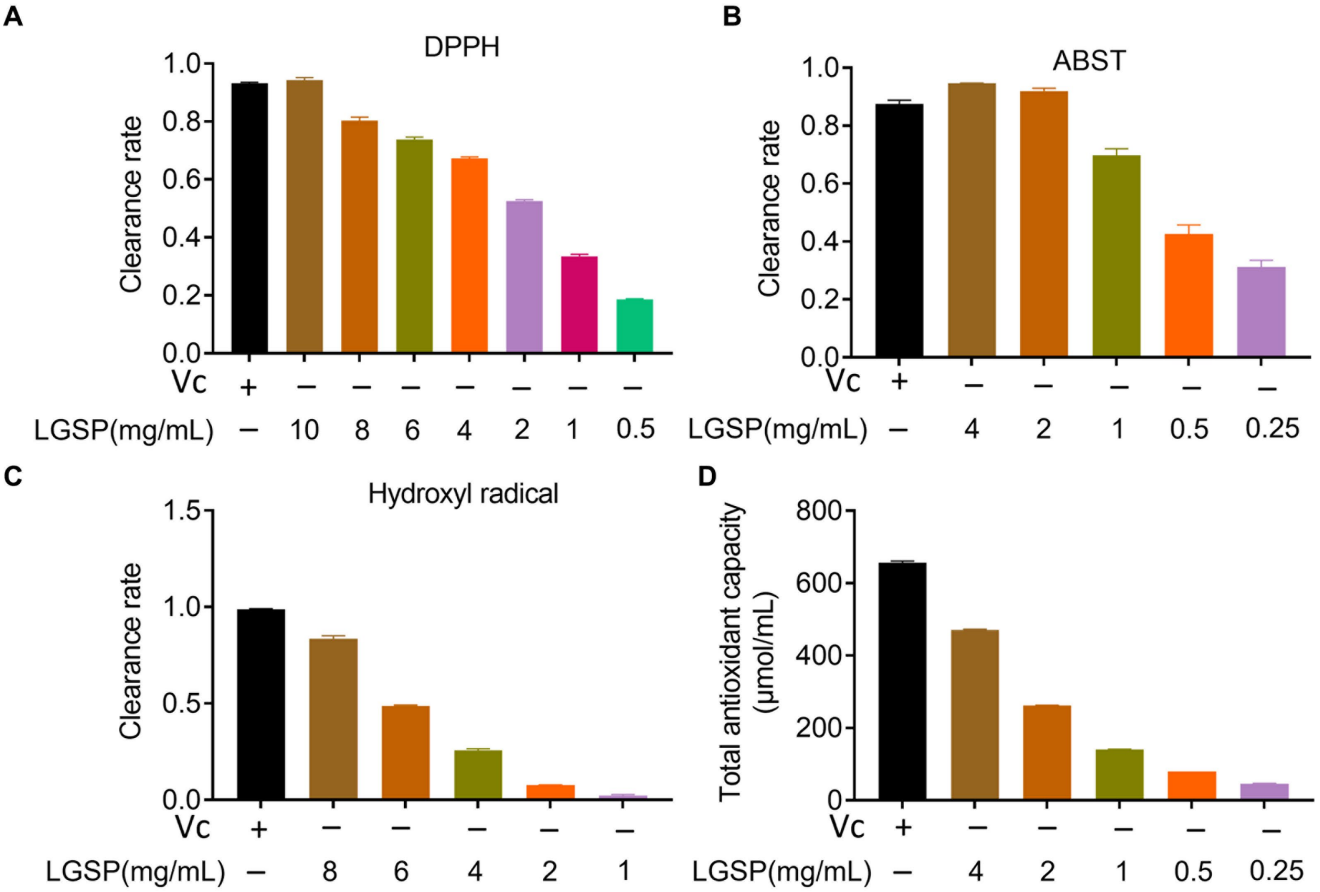
Antioxidant activity of different concentrations of LGSP. **(A)** Scavenging activity on DPPH radicals; **(B)** Scavenging activity on ABTS radicals; **(C)** Scavenging activity on hydroxyl radicals; **(D)** Total antioxidant capacity. Vitamin C was used as a positive control.

The anti-oxidation activity of LGSP was determined by DPPH, ABTS, hydroxyl radical scavenging capacity and total antioxidant capacity. The DPPH radical, characterized by a lone electron, exhibits notable stability despite its status as a free radical. Reacting with a scavenger, the solution undergoes a color change. (25). As can be seen in Figure 7A, the scavenging efficiency of LGSP on DPPH free radicals increased in direct proportion to the polysaccharide concentration, ranging from 1 to 10 mg/mL., and at a concentration of 10 mg/mL, the DPPH free radical scavenging rate reached 94.27%, slightly surpassing that of vitamin C (Vc) at 1 mg/mL.

ABTS has been used to evaluate the antioxidation ability of the substance (26). As displayed in Figure 7B, the ABTS free radical scavenging activity of LGSP was also positively correlated with dose. At the concentration was 4 mg/mL, the scavenging rate reached 94.66%, surpassing that of Vc at 0.05 mg/mL. These data indicate that LGSP exhibited significant efficacy in scavenging ABTS free radicals.

Among reactive oxygen species, hydroxyl radicals are very active, which may cause genetic alterations in cells and promote disease or cell death (27). As shown in Figure 7C, in the range of concentrations of 1–8 mg/mL, the scavenging ability of LGSP was gradually decreased. At the concentration of 8 mg/mL, its scavenging rate reached 82.97%, which was slightly lower than that of Vc at 10 mg/mL.

The total antioxidant capacity represents the level of various antioxidant substances in the system. As shown in Figure 7D, similarly, with the increase of LGSP concentration, total antioxidant capacity increased. At a concentration of 8 mg/mL, its total antioxidant capacity was 471.19 μmol/mL.

#### Anti-inflammatory effect

**Figure 8:**
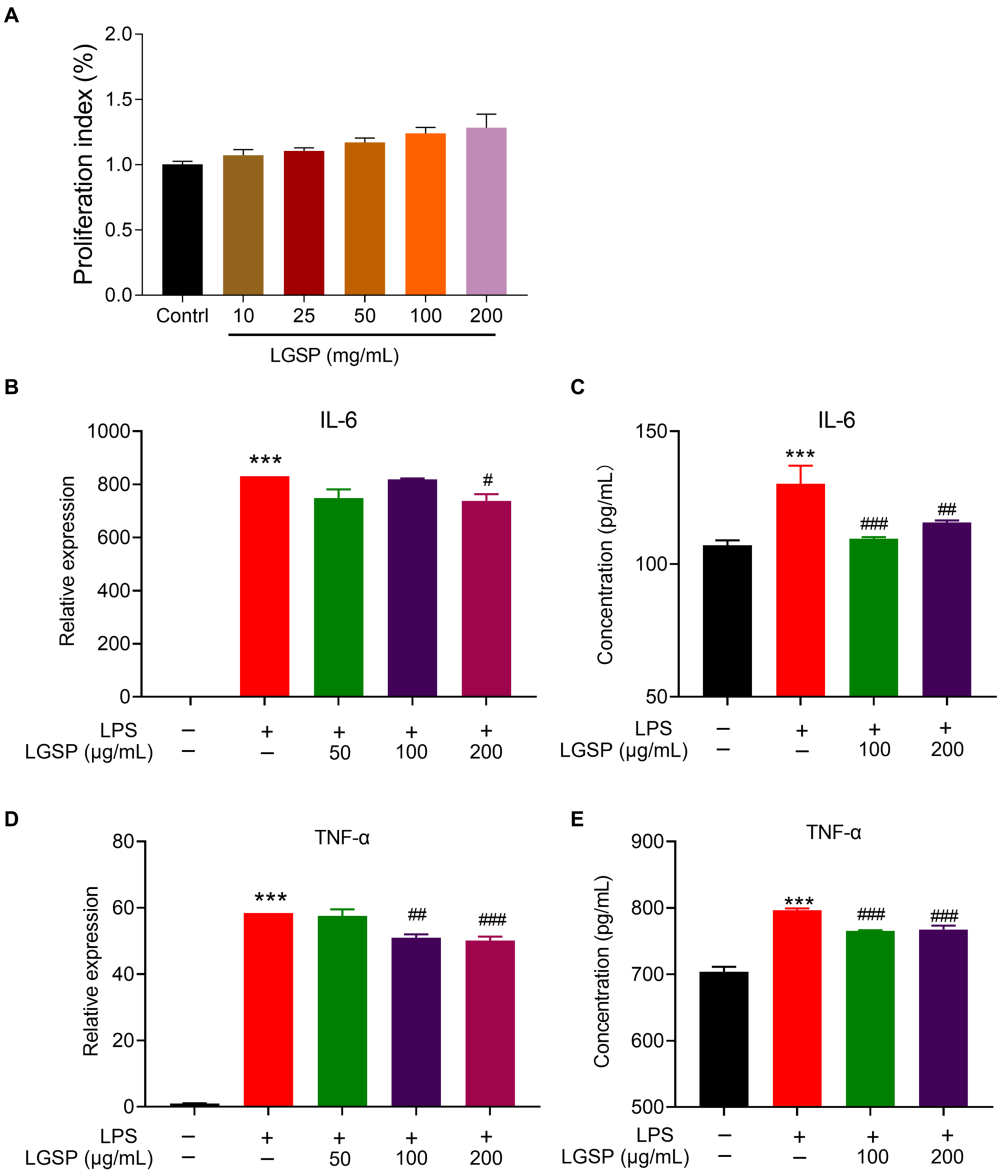
Anti-inflammatory effects of LGSP in LPS-induced RAW 264.7 cells. **(A)** Effects of LGSP on cell viability (24 h) of RAW 264.7 cells determined using MTT assays. **(B)** The mRNA expression of *IL-6* in LPS-induced RAW 264.7 cells. **(C)** The concentration of IL-6 in culture supernatant of LPS-induced RAW 264.7 cells detected by ELISA kit. **(D)** The mRNA expression of *TNF-α* in LPS-induced RAW 264.7 cells. **(E)** The concentration of TNF-α in culture supernatant of LPS-induced RAW 264.7 cells detected by ELISA kit. ^***^p < 0.001 vs. blank group; ^#^*p* < 0.05, ^##^*p* < 0.01, ^###^*p* < 0.001 vs. LPS alone group.

The effect of LGSP on cell viability of macrophages is analyzed by MTT assay before evaluating anti-inflammatory effect of LGSP. The result showed that LGSP was non-cytotoxic to macrophages at dose range of 10–200 μg/mL (Figure 8A).

RAW 264.7 cells were further stimulated by LPS to establish the experimental inflammation model. The relative expression levels of pro-inflammatory cytokines (IL-6 and TNF-α) in cells stimulated by LPS were detected by qPCR and ELISA kits. As shown in Figures 8B–D, the expression levels of these pro-inflammatory cytokines in LPS-induced RAW 264.7 cells were significantly higher than that in the non-LPS-induced group, suggestive of successfully establishment of the inflammatory cell model. Remarkably, LGSP treatment significantly inhibited both the gene and protein expression levels of IL-6 (Figures 8B,C) and TNF-α (Figures 8D,E) in RAW 264.7 cells in a dose-dependent manner, especially at the concentration of 100 and 200 μg/mL.

The above results indicated that LGSP had an anti-inflammatory activity on LPS-stimulated macrophages.

#### Prebiotic activity

Three strains of probiotics (*Lactobacillus*, *Bifidobacterium bifidum* and *B. adolescentis*), which have been demonstrated to alleviate liver dysfunctions, were used in this study (28, 29).

**Figure 9:**
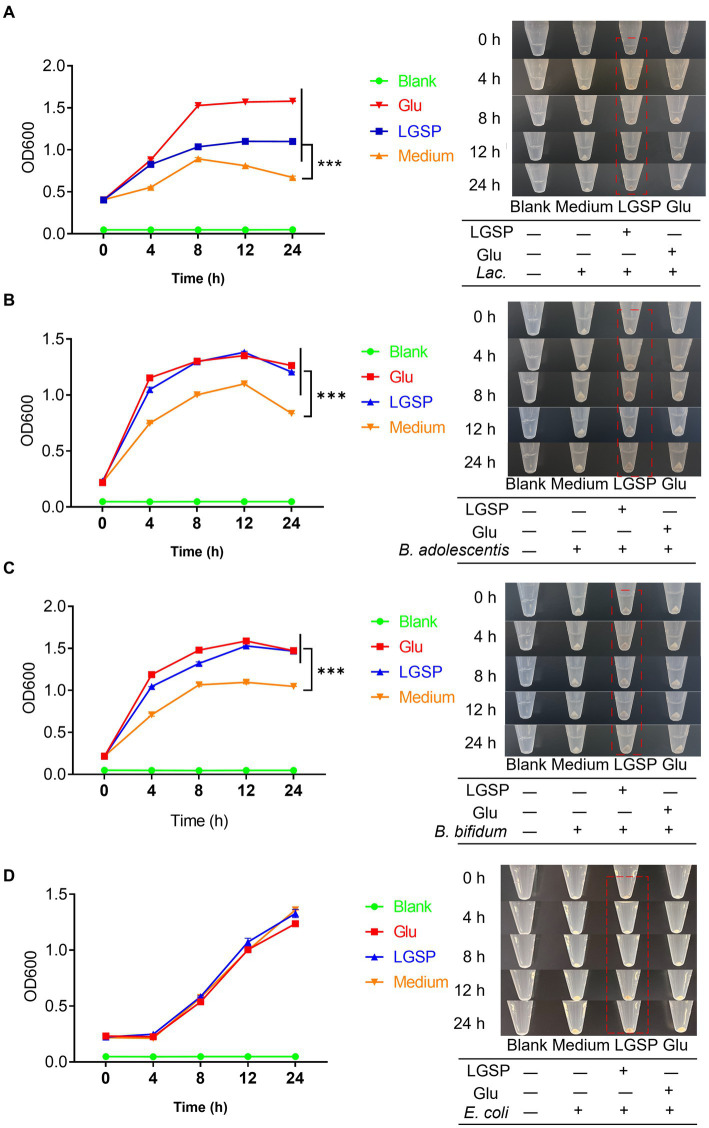
LGSP promoted the growth of three probiotic bacteria strains. **(A)** Growth curve of *Lactobacillus* (*Lac.*); **(B)** Growth curve of *Bifidobacterium adolescentis* (*B. adolescentis*). **(C)** Growth curve of *Bifidobacterium bifidum* (*B. bifidum*). **(D)** Growth curve of *Escherichia coli* (*E. coli*). Glucose (Glu) was used as positive control. ^***^p < 0.001 vs. Medium group.

Carbon sources were important for bacterial growth. To avoid interference of glucose, glucose-free medium was used for study. The results showed that supplementation of glucose-free medium with glucose or LGSP significantly promoted the growth of *Lactobacillus* (Figure 9A), *B. adolescentis* (Figure 9B) and *B. bifidum* (Figure 9C) when compared to the Medium group (using glucose-free medium only). Notably, for the growth of *Lactobacillus* (Figure 9A), the promoting effect of LGSP was smaller than that of glucose, while for the growth of *B. adolescentis* (Figure 9B) and *B. bifidum* (Figure 9C) the promoting effect of LGSP was comparable to that of glucose. The bacterial precipitation images at different time points also showed the same trend as the growth curve. In contrast, *in vitro* incubation experiments with *E. coli*, LGSP did not show an *in vitro* proliferative effect compared to the Medium group (Figure 9D). These results indicate that *Lactobacillus*, *B. bifidum* and *B. adolescentis* could ferment and use LGSP as a carbon source to support their proliferation *in vitro*, and LGSP displayed a prebiotic effect.

## Discussion

In this study, for the first time, we provided a comprehensive understanding on the chemical compositions and structural characteristics of LGSP through performing multiple-fingerprint analysis. Moreover, the anti-oxidant, anti-inflammatory and prebiotic effects of LGSP were revealed.

Quality control of polysaccharides poses a significant bottleneck in the development of novel polysaccharide-based pharmaceuticals. As a macromolecular substance, polysaccharides have a multi-level structure like proteins, which makes it difficult to evaluate their quality by content determination alone or by a specific spectrum (30). Recently, fingerprinting has been used as the preferred quality control method for complex natural polysaccharides due to its efficiency and convenience. From the aqueous extract of *Astragalus membranaceus*, Wang et al. purified polysaccharides, and the quality control of the polysaccharides was carried out by assessing polysaccharide content, monosaccharide composition and molecular weight (31). Jiang et al. isolated polysaccharides from sea cucumbers by anion exchange and gel permeation chromatography (32). HPSEC, PMP-HPLC, FT-IR spectroscopy, gas chromatography–mass spectrometry (GC–MS) and NMR spectroscopy were used to characterize the preliminary structure, and the results showed that the molecular weight of the polysaccharide was 9.3 × 10^5^ Da and the polysaccharide was composed of Glc, Gal, Ara and GlcA, with the major backbone of (1 → 4)-linked α-Glc*p*, (1 → 6)-linked β-Glc*p*, and (1, 4 → 6)-linked β-Glc*p* (32). Generally, polysaccharides are mainly controlled by molecular weight distribution, the types and proportions of constituent sugars, the types of characteristic functional groups, and the determination of characteristic glycosidic bond types and contents. In this study, LGSP contained two molecular weight distribution peaks, which were divided into fraction 1 and 2, with the average molecular weight of (6.569 ± 0.12) × 10^4^ Da, and (4.641 ± 0.30) × 10^4^ Da. LGSP was composed of 8 monosaccharides, including Man, Rha, Ara, Glc, Xyl, Gal, GlcA, GalA, with GalA and Glc representing the highest composition followed by Rha and Gal. FT-IR analysis suggested the presence of uronic acid, pyranonose and methoxy groups. NMR analysis found that HG, RG-I and α-1,4-glucan were present in LGSP. Quantitative analysis showed that LGSP yields 17.94 ± 0.28 mg/mL from LGS, with a protein content of 3.03% and total uronic acid content of 22.02%. Notably, batch-to-batch analysis of LGSP indicated that the quality of different batches of LGSP were generally consistent. The quality control research system established is thus considered complete and reliable.

The antioxidation activity of polysaccharides is related to the capacity to scavenge free radicals (24, 33). It is worth noting that the activity of polysaccharides is directly determined by the polysaccharide backbone and glycosidic bond type. Studies showed that the smaller the molecular weight of the polysaccharide, the higher the activity. The probable reason for this is that low-molecular-weight polysaccharides have more reducing ends and can bind better to free radicals (34–36). In addition to molecular weight, uronic acid, fucose, galactose, and galacturonic acid have also been identified as significant contributors to antioxidant activity. High composition of uronic acid in polysaccharides may lead to activation of isomeric hydrogen atoms to scavenge free radicals (37–39). In this study, the good free radical scavenging capacity of LGSP may be attributable to the relatively low molecular weight (10^4^ Da) and relatively high molar ratio of GalA (25.05%) and Gal (11.32%).

Macrophages are widely distributed and are important cells associated with inflammation, which are critical for immune stimulation and pathogen phagocytosis (40). Macrophages can differentiate into M1 type induced by LPS and generate the pro-inflammatory TNF-α, IL-1β and IL-6 (41). Numerous naturally occurring active polysaccharides demonstrate anti-inflammatory effects through modulating the secretion of inflammatory factors (42). The anti-inflammatory effect of polysaccharides is closely associated with the structural properties, and the content of galacturonic acid may directly affect their anti-inflammatory activity (43). In this study, it is found that LGSP has good anti-inflammatory activity by suppressing LPS-induced macrophage activation via inhibiting *TNF-α*, *IL-1β* and *IL-6*.

Polysaccharides with specific structures have good prebiotic activity (44). Increased probiotics may inhibit the systemic level of pro-inflammatory factors by correcting the imbalance of intestinal microbiota, increasing the proportion of probiotics, promoting the repair of intestinal wall and mucous membrane, inhibiting translocation of harmful bacterial metabolites (45, 46). Intestinal probiotics can decrease the lipid burden of the liver by regulating the intestinal microbial community, affecting the production of alcohol metabolites and the absorption of nutrients in the gut (47). Intestinal probiotics play a critical role in preserving immune balance within gut-liver axis, and their anti-inflammatory modulates the host’s immune system, improving alcohol-related liver damage and hepatic oxidative stress (48, 49). Previously, *Bifidobacterium* have been shown to increase glutathione peroxidase (GSH-Px) and glutathione (GSH) levels, reduce liver oxidative stress, while also reducing the expression of anti-inflammatory factors, reducing liver damage caused by ALD and ALD-associated intestinal dysbiosis (50, 51). In addition, *Lactobacillus* intake attenuates hepatic steatosis and liver injury, significantly reduces serum alanine aminotransferase (ALT) and aspartate transaminase (AST) levels, and ameliorates hepatic lipid droplet accumulation (28). Interestingly, *Lactobacillus* also has the effect of regulating intestinal bacteria (28). Therefore, modulating gut microbiota by applying probiotics such as *Bifidobacterium* and *Lactobacillus* has been regarded as promising therapeutic strategy for handling ALD and other liver diseases. In this study, we found that LGSP could remarkably promote the growth of *Bifidobacterium* and *Lactobacillus*, displaying prebiotic effect. The results provide evidence for understanding the polysaccharides as an active fraction of LGS to treat ALD. Furthermore, as the predominant pectin polysaccharide found in plants, HG-type pectin polysaccharides have the functions of regulating the intestinal microbiome and modulating the secretion of anti-inflammatory factors (52, 53). The finding that LGSP contains high content of GalA may suggest the potential association of typical structure of LGSP with its prebiotic effect. Although the starch fraction of LGS was removed during the extraction of LGSP, resistant starch (NMR signals showing T-α-D-Glc as well as 1,4-α-D-Glc) has been detected in LGSP. Resistant starch has also been investigated with prebiotic effect. Thus, the resistant starch in LGSP may also contribute to its prebiotic effect.

The main limitation of current study is that the anti-inflammatory and prebiotic effects of LGSP have not been validated *in vivo*. How these positive effects contribute to the anti-ALD effect of LGS warrants further investigation. Polysaccharides, polyphenols, flavonoids and others are mainly included in LGS, among which LGSP accounts for a high proportion (17.94 ± 0.28 mg/mL). The results of this study would help support the market use of LGS as a functional beverage and open up possible research directions (e.g., mechanisms of action and effective components) in the future.

## Conclusion

Multiple fingerprint profiling combined with composition quantification for quality evaluation of LGSP was established based on HPSEC, PMP-HPLC, FT-IR and NMR. The quality consistency of different batches of LGSP was assessed. The molecular weight distribution, monosaccharide contents and structural characteristics of LGSP were obtained. LGSP demonstrated anti-oxidant, anti-inflammatory and prebiotic effects, which correlated well with its structures. The results of current study would gain better understanding towards the structure of LGSP, provide guidance for the quality control, and support for the treatment of ALD by the active polysaccharide fraction of LGS.

## Data availability statement

The original contributions presented in the study are included in the article/supplementary material, further inquiries can be directed to the corresponding authors.

## Ethics statement

Ethical approval was not required for the studies on humans in accordance with the local legislation and institutional requirements because only commercially available established cell lines were used.

## Author contributions

SW: Writing – original draft, Formal analysis, Investigation, Validation. ML: Formal analysis, Investigation, Validation, Writing – original draft. LZ: Formal analysis, Investigation, Validation, Writing – original draft. TW: Formal analysis, Validation, Writing – review & editing. KW: Formal analysis, Validation, Writing – review & editing. JY: Formal analysis, Validation, Writing – review & editing. MT: Formal analysis, Writing – review & editing. YZ: Formal analysis, Writing – review & editing. JS: Formal analysis, Writing – review & editing, Validation. FD: Formal analysis, Validation, Writing – review & editing. YC: Formal analysis, Validation, Writing – review & editing. SD: Formal analysis, Validation, Writing – review & editing. ZX: Writing – review & editing, Resources. MW: Resources, Writing – review & editing, Project administration. ZL: Writing – review & editing, Conceptualization, Funding acquisition, Supervision. XW: Conceptualization, Funding acquisition, Supervision, Writing – review & editing, Project administration, Writing – original draft.

## Glossary

ALD: alcoholic liver disease
Ara: arabinose
ALT: alanine aminotransferase
AST: aspartate transaminase
BSA: bovine serum albumin
DMSO: dimethyl sulfoxide
FBS: fetal bovine serum
FT-IR: fourier transform infrared spectroscopy
Gal: galactose
GalA: galacturonic acid
Glc: glucose
GlcA: glucuronic acid
GSH: glutathione
GSH-Px: glutathione peroxidase
HG: homogalacturonic acid
HPAEC-PAD: high performance anion exchange chromatography-pulsed amperometric detector
HPSEC: high-performance size exclusion chromatography
LGS: Liuweizhiji Gegen-Sangshen beverage
LGSP: LGS polysaccharide
LPS: lipopolysaccharide
Man: mannose
MR: molar ratio
MTT: 5-diphenyltetrazolium bromide
NMR: nuclear magnetic resonance
OD: optical density
PMP-HPLC: 1-phenyl-3-methyl-5-pyrazolone-HPLC
Rha: rhamnose
RG-I: rhamnosegalacturonic acid glycans I
SD: standard deviation
TFA: trifluoracetic acid
UHPLC: ultra-high performance liquid chromatography
Xyl: xylose
